# Novel enhancer mediates the *RPL36A-HNRNPH2* readthrough loci and *GLA* gene expressions associated with fabry disease

**DOI:** 10.3389/fgene.2023.1229088

**Published:** 2023-12-13

**Authors:** Mohammed A. Al-Obaide, Saimul Islam, Ibtisam Al-Obaidi, Tetyana L. Vasylyeva

**Affiliations:** Department of Pediatrics, Texas Tech University Health Sciences Center, Amarillo, TX, United States

**Keywords:** fabry disease, α-Gal A, enhancer, lncRNA, readthrough locus, bioinformatic

## Abstract

Fabry disease (FD) is a rare genetic condition caused by mutations in the *GLA* gene, located on the X chromosome in the *RPL36-HNRNPH2* readthrough genomic region. This gene produces an enzyme called alpha-galactosidase A (α-Gal A). When the enzyme does not function properly due to the mutations, it causes harmful substances called globotriaosylceramide (Gb3) and globotriaosylsphingosine (lyso-Gb3) to build up in the body’s lysosomes. This accumulation can damage the kidneys, heart, eyes, and nervous system. Recent studies have shown that the *RPL36A-HNRNPH2* readthrough loci, which include *RPL36A* and *HNRNPH2* genes, as well as the regulatory sequence known as the *GLA-HNRNPH2* bidirectional promoter, may also play a role in FD. However, the involvement of enhancer RNAs (eRNAs) in FD is still poorly understood despite their known role in various diseases. To investigate this further, we studied an *RPL36A* enhancer called GH0XJ101390 and showed its genomic setting in the *RPL36-HNRNPH2* readthrough region; the eRNA is rich in Homotypic Clusters of TFBSs (HCTs) type and hosts a CpG Island (CGI). To test the functional correlation further with *GLA*, *RPL36A*, and *HNRNPH2*, we used siRNAs to knock down GH0XJ101390 in human kidney embryonic cells 293T. The results showed a significant decrease in *RPL36A* and *GLA* expression and a non-significant decrease in *HNRNPH2* expression. These findings could have important implications for understanding the regulatory mechanisms of GH0XJ101390 and its potential role in FD. A better understanding of these mechanisms may improve diagnostic and therapeutic methods for FD, which could ultimately benefit patients with this rare condition.

## Introduction

Fabry disease (FD, OMIM#301500) is a rare X-linked lysosomal storage disease. Deficiency in the activity of the α-Gal A enzyme encoded by the *GLA* gene, caused by pathogenic mutations that can be found on ClinVar—NCBI and have been identified in patients with Fabry disease (FD), leads to the accumulation of globotriaosylceramide (Gb3) and globotriaosylsphingosine (lyso-Gb3) in various cells throughout the body. These accumulations are associated with the development of FD (Schiffmann et al., 2000; Ortiz et al., 2018). This condition can cause damage to the kidneys, heart, and nervous system, and patients may experience various warning signs and clinical symptoms (Tuttolomondo et al., 2021). Individuals with this condition may experience a range of symptoms, including skin lesions (angiokeratomas), eye abnormalities (corneal verticillate), reduced sweating (hypohidrosis), ringing in the ears (tinnitus), gastrointestinal issues, premature aging, and other related ailments. (Dutra-Clarke et al., 2020; Burand and Stucky, 2021; Kurschat, 2021; Lo Curto et al., 2021). It is becoming increasingly evident that FD is a complex condition, as the effects of the buildup of lysosomal Gb3 and lyso-Gb3 are not yet fully comprehended. Recent findings suggest that the disease’s seriousness and the effectiveness of treatment cannot be solely attributed to the accumulation of Gb3 and lyso-Gb3 (Ortiz et al., 2018; Bichet et al., 2021; Tuttolomondo et al., 2021). Intriguingly, Ensembl (ensembl.org) and Gene-NCBI (ncbi.nlm.nih.gov/gene) databases note the potential implication of *RPL36A*-*HNRNPH2* readthrough loci in FD. The *RPL36*-*HNRNPH2* readthrough genomic region is located on the forward strand of chromosome X, while *GLA* is found on the opposite, reverse strand of the readthrough locus. According to recent experimental data, *RPL36A* and *HNRNPH2* are suggested to be involved in FD, as reported by Al-Obaide et al., in 2021 and 2022.

While mutations in the *GLA* gene’s a-galactosidase-A enzyme are considered the primary cause of FD, it is also possible that mutations in noncoding genomic regions that make up 98%–99% of the human genome could have a role in the disease. The ncRNA plays an important role in regulating gene expression, and even enhancers and promoters are considered part of it (Statello et al., 2021). In particular, enhancer RNAs (eRNAs) are essential for controlling gene expression and determining cell lineage specificity (Kim et al., 2010; Arnold et al., 2020). However, when these regulatory sequences malfunction, they can be linked to cancer and other age-related diseases, making targeted cancer therapy a potential treatment option (Kim et al., 2010; Chen et al., 2018; Zhang et al., 2019; Arnold et al., 2020; Tang et al., 2020; Richart et al., 2021; Liang et al., 2022; Zhang et al., 2022). Mutations in enhancers have also been implicated in various diseases (Claringbould and Zaugg, 2021). Interestingly, research has shown that promoters with enhancer activity can also regulate gene expression (Dao and Spicuglia, 2018; Medina-Rivera et al., 2018; Tippens et al., 2018). As a result, understanding and utilizing ncRNA has become increasingly important for potential diagnostic and therapeutic applications in the medical field. Recent studies have revealed a connection between ncRNA and FD. For instance, pathogenic variants have been discovered in the *GLA-HNRNPH2* bidirectional promoter (BDP), as reported by Al-Obaide et al., in 2021. Additionally, Lo Curto et al., 2021 found that premature aging in FD patients is correlated with miR-126-3p and the accumulation of lyso-Gb3 in FD patients.

The GeneHancer database (Fishilevich et al., 2017) displays enhancers, promoters, and promoters with enhancer activities and their target genes. The GeneHancer shows a predicted sequence called GH0XJ101390 with enhancer and promoter features and potential regulatory activity at the 5′-side of the *RPL36A* gene. The GeneHancer predicted that GH0XJ101390 can target several genes, including the *RPL36A* and *GLA* genes. This study shows that the GH0XJ101390 sequence hosts unreported CpG Island (CGI) and Homotypic Clusters of TFBSs (HCTs). To date, no experimental studies have been conducted to determine how the GH0XJ101390 enhancer affects the expression of the *RPL36A*-*HNRNPH2* readthrough loci, *RPL36A* and *HNRNPH2,* and the *GLA* locus. We report that using siRNA knockdown of GH0XJ101390 decreases the *GLA* and *RPL36A* expression. Our results may suggest the potentially critical role of GH0XJ101390 enhancer in FD, considering its role in regulating *GLA* and *RPL36A* loci. The reported results could aid in better understanding the role of GH0XJ101390 in the broad range of clinical symptoms of FD, leading to improved diagnosis and treatment approaches for FD.

## Materials and methods

### Database search and analyses of GH0XJ101390 enhancer and related genes

The GeneHancer database, which is part of the GeneCards Suite, includes the predicted GH0XJ101390 enhancer. GeneHancer links enhancers to genes by using four methods, including tissue co-expression correlation between genes and enhancer RNAs, enhancer-targeted transcription factor genes, expression quantitative trait loci for variants within enhancers, and capture Hi-C, which is a promoter-specific genome conformation assay. GeneHancer’s combinatorial likelihood-based scores for enhancer-gene pairing are based on individual scores from each of these four methods, as well as gene-enhancer genomic distances (Fishilevich et al., 2017). The GH0XJ101390 sequence was downloaded from the UCSC Genome Browser bioinformatics website, which provides sequence data and annotations (Karolchik et al., 2003; Lee et al., 2022). Even though GeneHancer displays 211 TFBSs, it does not reveal the presence of Homotypic Clusters of TFBSs (HCTs) in the GH0XJ101390 sequence. To identify HCTs, we employed two bioinformatics tools - the JASPAR and AnimalTFDB search tools. JASPAR is supported by several open-source software tools and Application Programming Interfaces (APIs) built using various programming languages like Python/Biopython and R/Bioconductor (Castro-Mondragon et al., 2022). AnimalTFDB uses the HMMER package to search and predict transcription factors (TFs) and DNA-binding domain (DBD). Moreover, the transcription factor binding sites (TFBS) are extracted from the HOCOMOCO, TRANSFAC, JASPAR, and CISBP databases (Zhang et al., 2012; Shen et al., 2023). We utilized these tools to identify HCTs in the GH0XJ101390 sequence. We utilized the default set parameters of “EMBOSS Cpgplot” to identify the CpG Island (CGI). The search parameters we used were: Observed/Expected ratio >0.60, %G + %C > %50, and CGI length >200. To align DNA sequences, we used two alignment tools: EMBOSS Needle for end-to-end alignment and EMBOSS Matcher to identify similar regions within the sequences. We referred to several online public genomics databases including NCBI-Gene, GeneCards, UCSC Genome Browser, and Ensembl Genome Browser to define the genomic setting of GH0XJ101390 to *RPL36-HNRNPH2* readthrough, *GLA*, *RPL36A*, and *HNRNPH2* loci. Data mining and bioinformatics analysis were conducted from January 2021 to September 2022. Supplementary Table S1 includes URLs for genomics databases and bioinformatics tools used in the study.

### Human cell line 293T [HEK-293] and RNA extraction

We acquired the Human Embryonic Kidney 293T cell line (ATCC^®^ CRL-1573™) from the American Type Culture Collection (ATCC). The cells were grown in Eagle’s Minimum Essential Medium (EMEM), ATCC catalog number 30–2003, with 10% heat-inactivated FBS (ATCC^®^ 30-2020TM), 100 U/mL penicillin, and 100 μg/mL streptomycin. The incubation occurred in a humidified incubator with 5% CO2 at 37°C. To extract RNA from 293T cells, we utilized a purification kit (cat. no. 48700) from Norgen Biotek Corp, Thorold, ON, Canada.

### siRNA transient transfections

The siRNA-27 kit (Catalog # SR304146) was purchased from OriGene, Rockville, MD, United States. The kit includes three siRNAs targeting specific sequences in the GH0XJ101390 and *RPL36A* transcript. The siRNA, identified as SR304146A-GGAACCUCCUAUAUACUUCCGUUUG, targets a specific site in the enhancer GH0XJ101390 transcript upstream of the *RPL36A* sequence. The siRNA SR304146C-GCUCACGCAAGCAUGGUUAACGUCC targets specific sequences in the GH0XJ101390 transcript and *RPL36A* transcript and untranslated transcript of exon-1 and translated exon-2 transcript. Meanwhile, SR304146B-GGACUUUCUGUAAGAAGUGUGGCAA targets the RL36A exon-2 (Supplementary Table S2, Supplementary Figure S1). The 293T cultured cells underwent transient transfection following the OriGene’s protocol. To introduce siRNA pool or the SR30004-scrambled negative control siRNA into the cells, we used the Roche X-tremeGENE siRNA transfection reagent and followed the manufacturer’s instructions, using a concentration of 10 nM. Four types of treatments were done on 293T cells. The first group was the control and remained untreated. The second group was treated with an X-tremeGENE transfection reagent, the third group was treated with the siRNA pool containing three different siRNAs, and the last group was treated with scrambled control siRNA. To prepare siRNAs and the transfection reagent, an Opti-MEM medium was utilized. The cells were cultured in EMEM with the addition of 10% heat-inactivated FBS. After 48 h of transfection, the cells were trypsinized and prepared for RNA extraction or Western blotting.

### Primer design and RT-qPCR analysis

We utilized the PrimerQuest Tool to design custom primers for *RPL36A*, *HNRNPH2*, and *GLA* (Supplementary Table S3). The Taq universal SYBR Green One-Step Kit (BIO-RAD) protocol was followed to perform RT-qPCR reactions in triplicate. The BIO-RAD iCylcer iQ system software quantified the reaction measurements. To analyze the expression of target genes *GLA*, *HNRNPH2*, and *RPL36A*, normalization and relative expression analysis were conducted using *HPRT1* as a reference gene (Al-Obaide et al., 2021; 2022). The Cq average value to determine the 2^−ΔΔCT^ to examine the relative gene expression data from real-time quantitative PCR (Livak and Schmittgen, 2001; Schmittgen and Livak, 2008).

### Western blotting

The protein levels of *GLA*, *HNRNPH2*, and *RPL36A* were assessed by Western blot analysis from three replicates. To perform the analysis, 293T cells were lysed using Pierce IP Lysis buffer from Pierce Biotechnology in Rockford, United States. The supernatants obtained after centrifugation were used for analysis, and the protein concentrations were determined using a BCA Protein Assay Kit (Pierce Biotechnology). Fifty micrograms of protein were separated on a 10% SDS-PAGE gel and then transferred to an Immobilon-P membrane (Millipore Co.) after blocking in 4% BSA solution. The blots were then incubated with one of the following primary antibodies (host: rabbit): α-galactosidase antibody (Invitogen, cat. # PA5-27349), RPL36A antibody (Invitogen, cat. # PA5-70719), anti-HNRNPH2 (Sigma, # A00956) and beta Actin antibody (Invitogen, cat. # PA5-85490) for 6 h. The blots were then incubated with goat anti-rabbit IgG, and horseradish peroxide conjugated. Positive bands were visualized by enhanced chemiluminescence (ECL, Amersham Co.). The Western blots were photographed, and the bands were quantitated by densitometry using a VersaDoc 5,000 imaging system. ImageJ software (imagej.nih.gov) was used for data analysis of the investigated protein products of *GLA*, *HNRNPH2*, and *RPL36A*.

### Statistical analysis

The siRNA experiments were conducted thrice, and each siRNA treatment was subjected to triplicate qPCR measurements. The Cq mean data from three experiments were used for 2^−ΔΔCT^ analysis. Microsoft Excel 365 was employed for data sorting, analysis, and drawing diagrams. The data has been presented as the mean ± SD. For analyzing two independent samples, a *t*-test was used, and a significant difference was considered at *p* < 0.05.

## Results

### The genomic setting of the *RPL36A*-*HNRNPH2* readthrough locus and the predicted GH0XJ101390 enhancer

**FIGURE 1:**
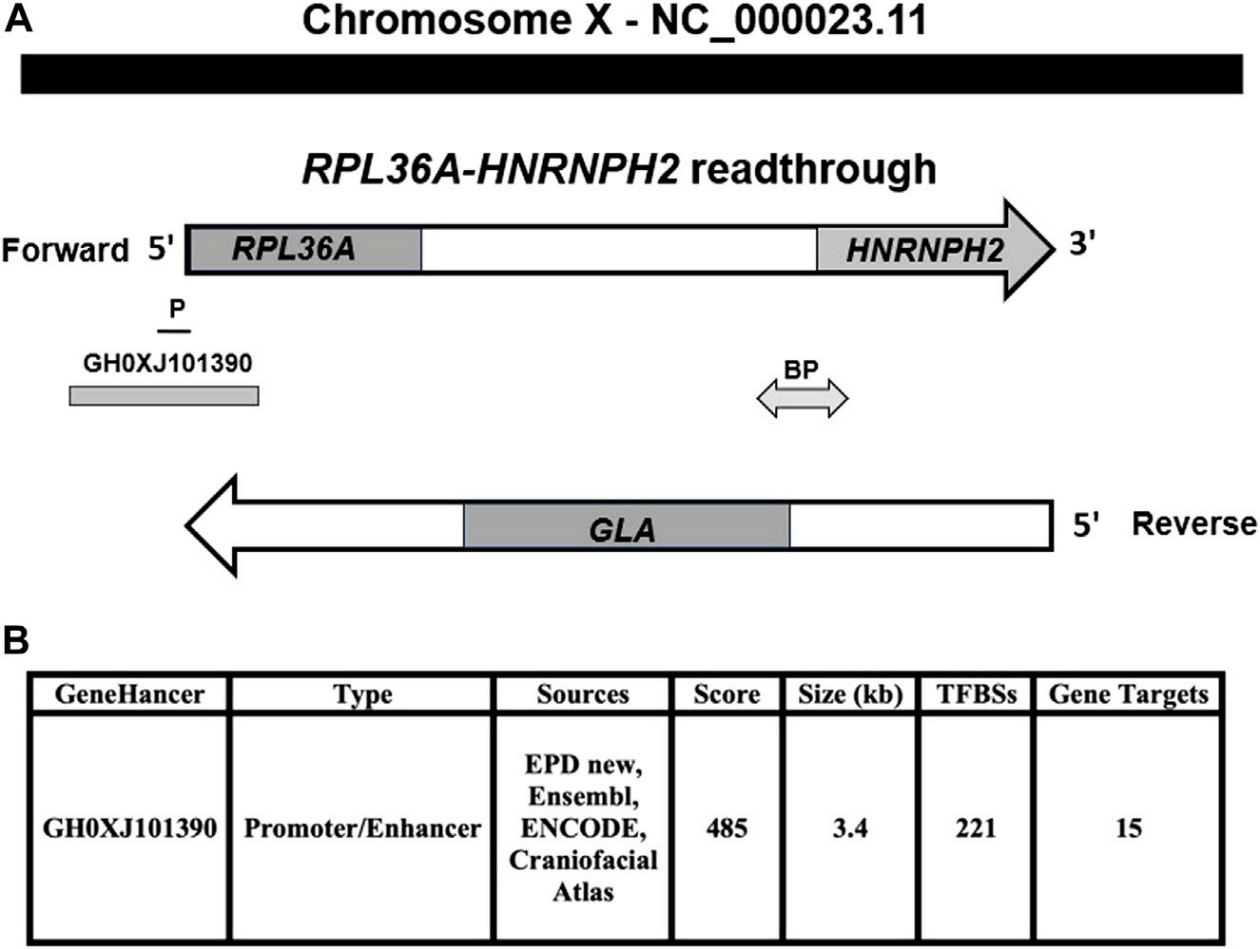
The genomic setting of GH0XJ101390 eRNA. **(A)** *RPL36A-HNRNPH2* readthrough genomic region located on the chromosome X forward strand, *RPL36A* upstream, *HNRNPH2* downstream, *GLA* gene at the reverse strand. **(B)** Genomic features of GH0XJ101390 enhancer and inferred number of target genes. GeneHancers are assigned a confidence score based on the number of sources, source scores, and TFBSs (from ENCODE). P: *RPL36A* promotor, BP: *GLA,* and *HNRNPH2* bidirectional promoter.

The read-through *RPL36A-HNRNPH2* locus is composed of two genes, the first is upstream, *RPL36A* coding ribosomal protein L36a with similarity to readthrough encoded protein, as shown by the matcher and needle alignment programs. The second gene is the downstream, coding heterogeneous nuclear ribonucleoprotein H2 gene, *HNRNPH2*. The *GLA* locus, which is implicated in FD, is mapped in between the *RPL36A* and *HNRNPH2* loci on the reverse strand of the *RPL36A*-*HNRNPH2* readthrough locus (Figure 1A). GeneHancer identifier affiliated with the GeneCards database shows an enhancer, GH0XJ101390, associated with regulating *RPL36A*, *GLA* genes, and 13 additional loci (Figure 1B). Our analysis revealed the GH0XJ101390 enhancer’s sequence occupies a genomic region at genomic coordinates (GRCh38): chrX: 101,390,257-101,393,641, upstream of *RPL36A*-*HNRNPH2* readthrough locus on the forward strand of the long arm of chromosome X (Figure 1A, Supplementary Figure S1A). Alignment analysis showed the enhancer sequence overlaps upstream the 5′-side of the *RPL36A* locus and downstream untranslated *RPL36A* exon-1, Intron 1, exon 2, and Intron 2; also, the GH0XJ101390 sequence overlaps the *RPL36A* promoter (Figure 1A). Supplementary Figure S1B shows the human *RPL36A* NM_021029.6 mRNA transcript used in the analysis to identify the *RPL36A* untranslated exon 1 and translated exon 2 in the genomic sequence of the GH0XJ101390 enhancer. Stergachis et al., 2013 showed that codons, which specify amino acids host transcription factors binding sites.

### *In silico* analysis of GH0XJ101390 sequence

**FIGURE 2:**
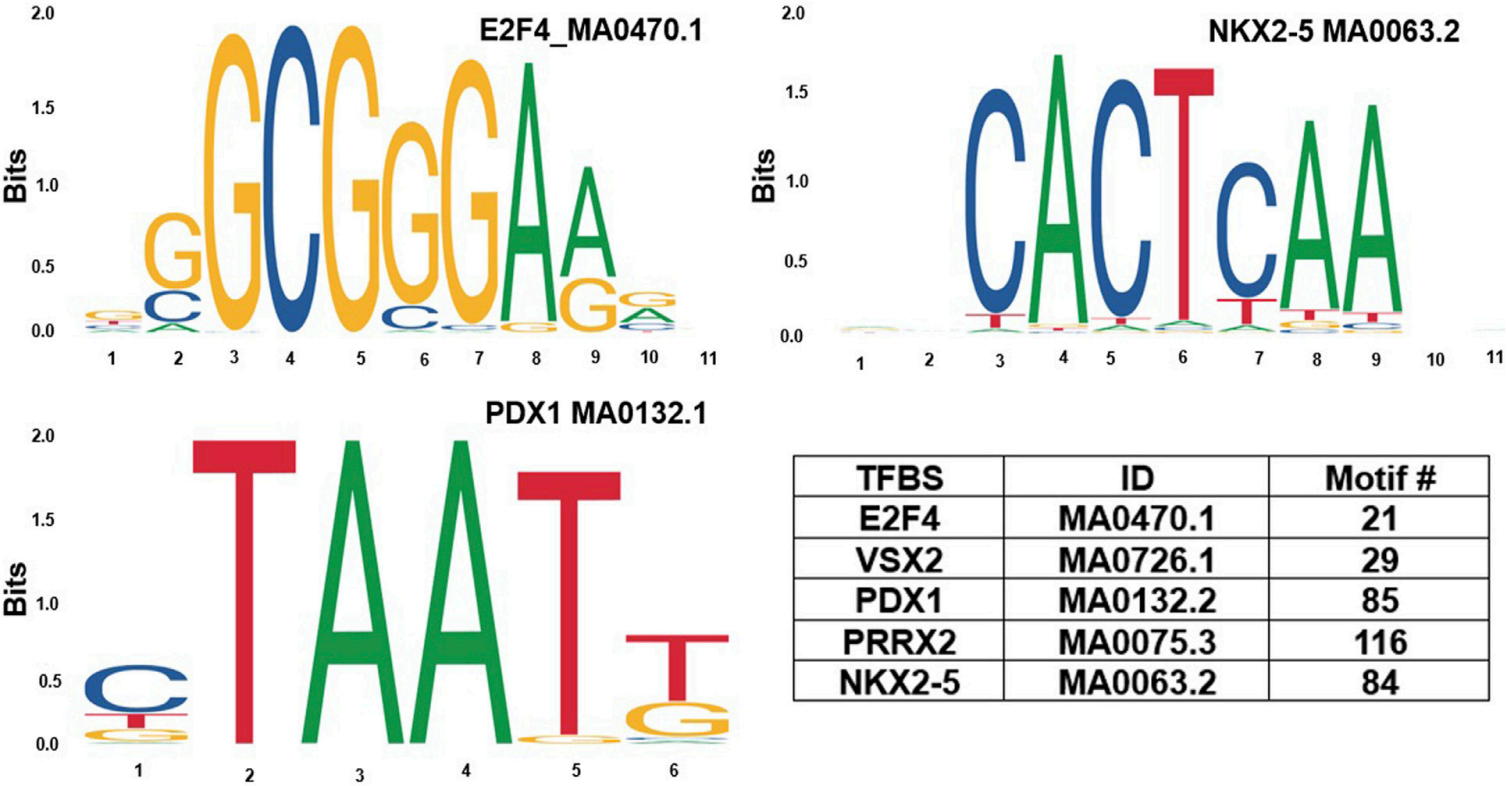
Examples of homotypic clusters of TFBSs (HCTs) profiles identified in the GH0XJ101390 eRNA sequence, and the ID and number of HCTs motifs identified in the GH0XJ101390 sequence.

**FIGURE 3:**
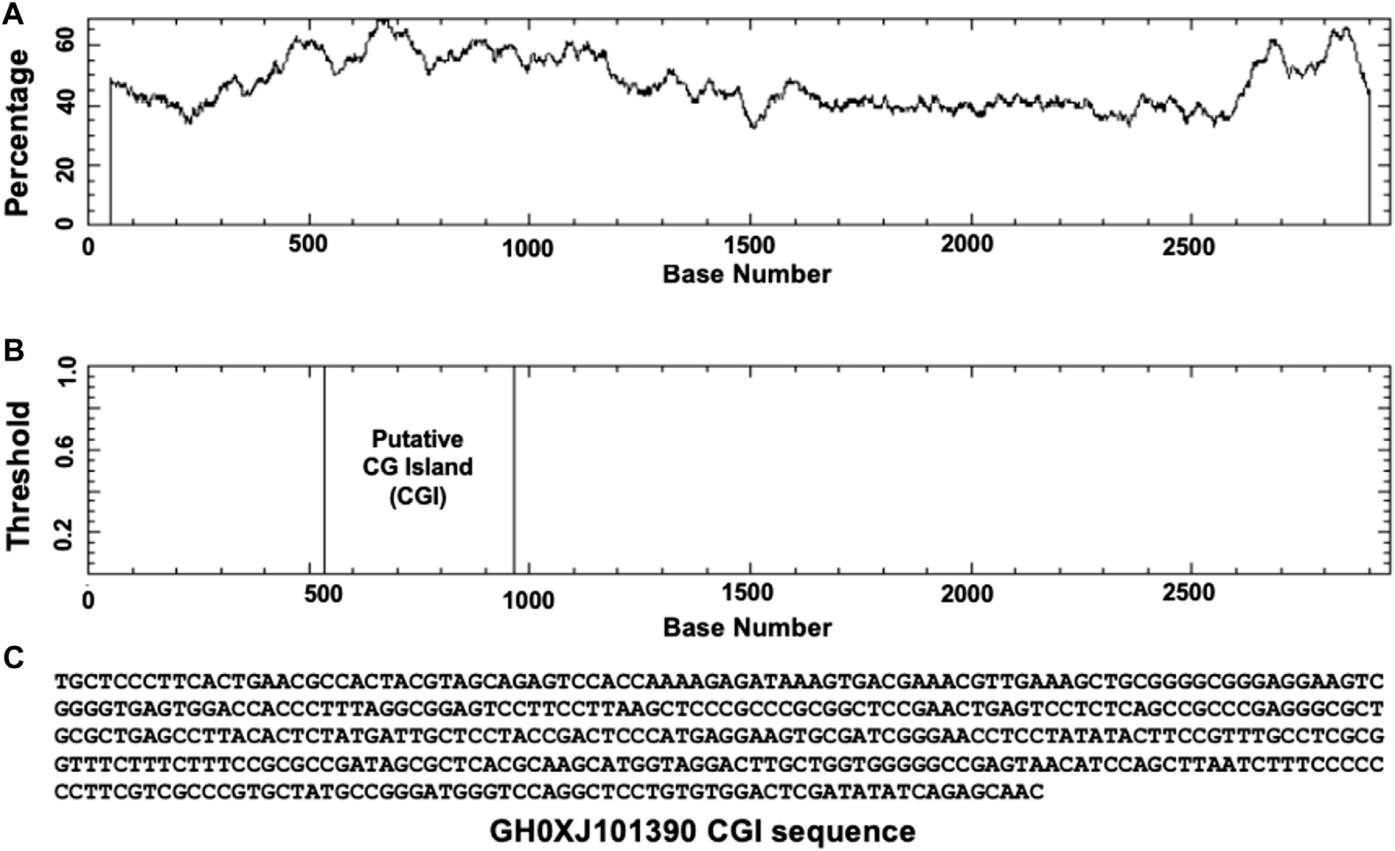
Identification of CGI in the GH0XJ101390 sequence. **(A)** percent C + percent G in the GH0XJ101390 sequence, **(B)** the position of CGI in the GH0XJ101390 nucleotide sequence, and **(C)** the identified CGI sequence.

Based on GeneHancer’s findings, the GH0XJ101390 spans 3.384 kb and contains 211 transcription factor binding sites (TFBSs). However, GeneHancer did not show any Homotypic Clusters of TFBSs (HCTs) in this enhancer, which are crucial components of enhancers (Gotea et al., 2010). Nonetheless, a bioinformatics analysis conducted in this study found eight HCTs types in the GH0XJ101390 sequence, which are POU3F2, NRF1, E2F4, TEF, VSX2, PDX1, PRRX2, and NKX2-5. Figure 2 provides examples of HCTs and their profiles. Furthermore, our analysis identified a CGI upstream of the GH0XJ101390 sequence. A CGI is a crucial part of an enhancer that helps to extend its long-range regulatory activity and control the responsiveness of its target genes (as discussed). The CGI sequence that we found is 428 bps in length and runs from nucleotide 537 to 964 along the GH0XJ101390 sequence, which is 3.384 kb. This CGI sequence is located at chrX: 101390737–101391163, as shown in Figure 3.

### Regulation of *GLA*, *RPL36A* and *HNRNPH2* expressions by GH0XJ101390 eRNA

**FIGURE 4:**
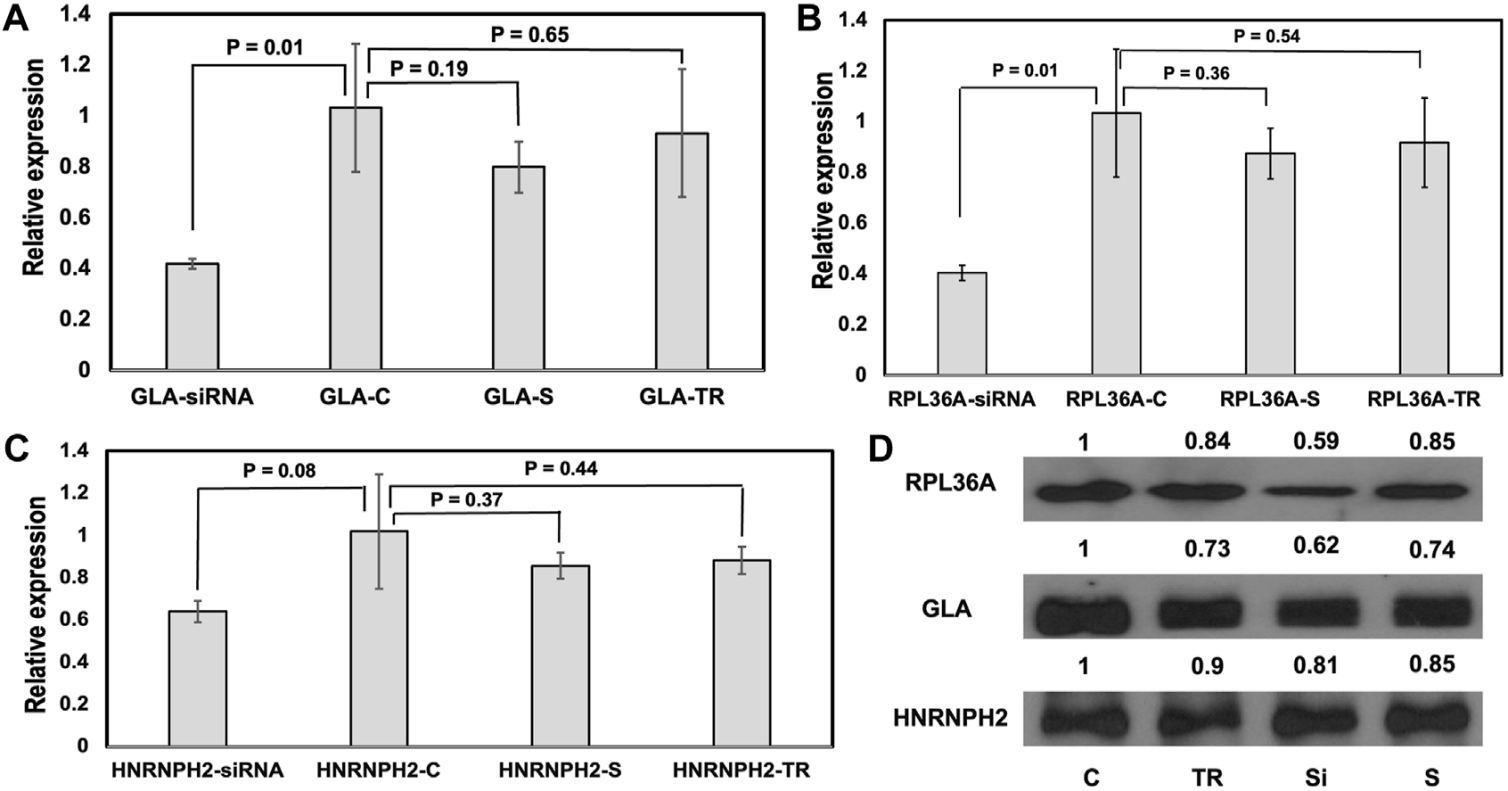
GH0XJ101390 influences the expressions of *GLA, RL36A*, and *HNRNPH2*. **(A–C)** RT-qPCR measured relative expressions of *GLA*, *RPL36A,* and *HNRNPH2* in 293T cells treated with siRNA compared to control. *p < 0.05, ns: *p* > 0.05. **(D)** Western blot analysis of RPL36A, GLA, and HNRNPH2 proteins following siRNA- GH0XJ101390 knockdown in 293T cell line. C: untreated cells, TR: treatment by transfection reagent, S: treatment by scrambled control siRNA, siRNA: treatment by siRNA pool.

As far as we know, there have not been any experimental studies that demonstrate the effect of GH0XJ101390 enhancer on the expression of *GLA, RPL36A,* and *HNRNPH2* genes. To explore the regulatory function of the GH0XJ101390, we conducted research on the human embryonic kidney 293T cell line. This cell line was chosen for its favorable characteristics in transfection. In order to determine the role of GH0XJ101390 in regulating *GLA,* and the other two loci *RPL36A* and *HNRNPH2*, we conducted a GH0XJ101390 siRNA silencing experiment on 293T cells using specific siRNAs that target the GH0XJ101390 sequence. After 48 h of transient transfection by siRNAs of 293T cells, RT-qPCR showed significant downregulation of *GLA* and *RPL36A* expressions, *p* < 0.05 (Figures 4A, B, Supplementary Table S4). This finding links the knocking down of GH0XJ101390 and *GLA* expression. On the other hand, a trend of non-significant decrease in *HNRNPH2* was also observed by RT-qPCR, *p* > 0.05 (Figure 4C). The Western blot data indicated a decrease in GLA and RPlA36A proteins but was not observed in the HNRNPH2 protein (Figure 4D).

## Discussion

Dysregulation of lncRNAs can lead to various diseases, including neurological and cardiovascular disorders (Esteller, 2011; Statello et al., 2021). Enhancers and promoters are examples of lncRNAs (Ørom and Shiekhattar, 2011; Ma et al., 2013; Kopp and Mendell, 2018; Han and Li, 2022; Mach and Giorgetti, 2023). Enhancer RNAs (eRNAs) are a specific type of lncRNA transcribed from enhancer regions of DNA sequences. They indicate gene expression, cell lineage specificity, and function or malfunction (Kim et al., 2010; Arnold et al., 2020). Thus, dysfunction of these regulatory sequences can result in various diseases (Izumi, 2016; Zhang et al., 2019; Gordevicius et al., 2021; Okabe and Kaneda, 2021; Zhang et al., 2022). Additionally, enhancers are linked to aging-related diseases (Chen et al., 2018). According to the findings of this study, the GH0XJ101390 eRNA may have a significant role to play in the expression of *GLA*. Although *GLA* is still a significant gene that impacts the clinical spectrum, disease progression, and treatments related to FD, there is growing suspicion that *GLA* pathogenic mutations alone may not be the sole cause of FD. Recent studies (Lo Curto et al., 2021; Tuttolomondo et al., 2021) suggest that FD may also occur due to the dysregulation of certain miRNAs such as miR-1307-5p and miR-126-3p levels, leading to progressive damage and premature aging of the kidneys, heart, and nervous system.

Through our study, we were able to identify the CGI present in the GH0XJ101390 sequence. The CGIs play a crucial role in enhancers, enhancing their long-range regulatory activity and controlling the responsiveness of target genes, as per Crispatzu et al. (2021). Since GH0XJ101390 modulates transcription, the identified CGI in the GH0XJ101390 sequence is likely involved in the long-range activity of GH0XJ101390, thus extending its function to *GLA* and *HNRNPH2* expressions. Our experiments carried out in human 293T cells, were designed to provide data about potential transcriptional control of the *RPL36A*, *GLA*, and *HNRNPH2* by GH0XJ101390 eRNA. The siRNA transient transfection experiments were conducted to knock down the GH0XJ101390 eRNA transcript(s), which resulted in a significant decrease in *RPL36A* and neighboring *GLA* expressions. However, *HNRNPH2* expression was only insignificantly decreased. We chose to carry out our experiments in human 293T cells since it is widely utilized in transfection experiments due to its high transfectivity, rapid growth rate, and ability to grow *in vitro* culture (Tan et al., 2021).

Though the presence and regulatory functions of enhancers are undisputed, there is still a need for more information about their mechanisms in regulating gene expression. Our present study indicates that the GH0XJ101390 enhancer is involved in the regulation of *RPL36A* and *GLA* genes. However, the precise mechanism that regulates the *GLA* and *RPL36A* loci is yet to be understood. Our study shows that the GH0XJ101390 sequence coincides with the *RPL36A* exon1 and exon2 sequences. This finding sheds light on the dual purpose that human codons serve, specifying both amino acids and transcription recognition sites (Stergachis et al., 2013; Waldrop et al., 2019).

To better understand the function of GH0XJ101390 eRNA, further studies are necessary. Specifically, we should investigate the impact of specific siRNA on the GH0XJ101390 eRNA sequence upstream of *RPL36A* in comparison to siRNA that knockdown GH0XJ101390 sequences downstream of the *RPL36A* 5′-UTR region. Additionally, examining the effect of GH0XJ101390 on the *RPL36A-HNRNPH2* readthrough locus is crucial. However, such a study presents a challenge due to the fact that the *RPL36A-HNRNPH2* transcript spans two genes, and the NCBI-Gene reports the *RPL36A-HNRNPH2* readthrough transcript produces a protein with similarity to the protein encoded by the upstream locus, *RPL36A*. As a result, further research is necessary to fully comprehend this critical genomic setting in readthrough transcript and protein products. In addition, it would be intriguing to explore how the secondary structure(s) of eRNAs affect their regulatory functions, as well as the impact of enhancer methylation on gene expression. It would also be worthwhile for future studies to investigate these matters in healthy individuals and compare them to GH0XJ101390 in FD patients.

To sum up, our study has uncovered an exciting possibility through analyzing the genomic setting of the GH0XJ101390 and experimental analysis. It seems that GH0XJ101390 may play a role in regulating the *GLA*. Furthermore, our bioinformatics analysis has identified homotypic clusters of TFBSs (HCTs) and CG Island (CGI) in the enhancer sequence of GH0XJ101390. This CGI appears to enhance the enhancer’s long-range regulatory activity, which in turn helps to modulate the expressions of *GLA*. This information can aid in understanding the underlying causes of Fabry disease and potentially may lead to the development of new diagnostic and treatment methods.

## Data Availability

The study’s original contributions are publicly available at [Link: https://www.ebi.ac.uk/biostudies/studies/S-BSST1240]. Supporting data and information accession number: S-BSST1240.
