# Supplementary material for: Novel enhancer mediates the RPL36A-HNRNPH2 readthrough loci and GLA gene expressions associated with fabry disease
Source: Front Genet. 2023 Dec 13;14:1229088. doi: 10.3389/fgene.2023.1229088 (PMC10753776; doi:10.3389/fgene.2023.1229088)

**List of the contents of this file**

1. **Supplementary Tables S1-S4**

**Table S1:** Web-based databases and bioinformatics tools used in this study.

| Database | URL |
| --- | --- |
| NCBI-Gene | <https://www.ncbi.nlm.nih.gov/gene/> |
| NCBI-Nucleotide | <https://www.ncbi.nlm.nih.gov/nuccore/> |
| GeneCards | <https://www.genecards.org> |
| ClinVar – NCBI | <https://www.ncbi.nlm.nih.gov/clinvar/> |
| The UCSC Genome Bioinformatics website | <https://genome.ucsc.edu> |
| UCSC Genome Browser | <https://genome.ucsc.edu> |
| Ensembl Genome Browser | <https://useast.ensembl.org/index.html> |
| Ensembl regulatory build | <https://useast.ensembl.org/info/genome/funcgen/index.html> |
| ENCODE | <https://www.encodeproject.org> |
| EPDnew | <https://epd.expasy.org/epd/> |
| EMBL-EBI | <https://www.encodeproject.org> |
| EMBOSS Matcher | <https://www.ebi.ac.uk/Tools/psa/emboss_matcher/> |
| EMBOSS Needle | <https://www.ebi.ac.uk/Tools/psa/emboss_needle/> |
| EMBOSS Cpgplot | <https://www.ebi.ac.uk/Tools/seqstats/emboss_cpgplot/> |
| JASPAR search tool | <https://jaspar.genereg.net> |
| AnimalTFDB | <http://bioinfo.life.hust.edu.cn/AnimalTFDB> |
| FANTOM5 atlas of active enhancers | <https://fantom.gsc.riken.jp> |
| VISTA Enhancer Browser | <https://enhancer.lbl.gov> |
| dbSUPER super-enhancers | <http://asntech.org/dbsuper/> |
| ImageJ | <https://imagej.nih.gov/ij/download.html> |

**Table S2.** The specific siRNA sequences used in the analysis.

| **siRNA**  **Code** | **siRNA sequence of exon 1** |
| --- | --- |
| **SR304146A** | **GGAACCUCCUAUAUACUUCCGUUUG** |
| **SR304146B** | **GGACUUUCUGUAAGAAGUGUGGCAA** |
| **SR304146C** | **GCUCACGCAAGCAUGGUUAACGUCC** |

**Table S3:** Designed primers for *RPL36A, GLA, HNRNPH2*, and reference gene.

| **Genes** | **Primers’ codes** | **Primer sequence** |
| --- | --- | --- |
| *RPL36A* | R-F | CGCTGAGCCTTACACTCTATG (Sense) |
|  | R-R | GGCGCGGAAAGAAAGAAAC (AntiSense) |
| *HNRNPH2* | H-F | AGTAGTTCTGGTCGTCGTCTA (Sense) |
|  | H-R | ACACACCAACCTCTAACGATAC (AntiSense) |
| *GLA* | G-F | AGGTTACCCGCGGAAATTTAT (Sense) |
|  | G-R | GAAACGAGGGCCAGGAAG (AntiSense) |
| *HPRT1* (reference gene) | HP-F | TGAGGATTTGGAAAGGGTGT (Sense) |
|  | HP-R | GAGCACACAGAGGGCTACAA (Antisense) |

**Table S4:** Unpaired t-test results P values of *GLA, RPL36A,* and *HNRNPH2* relative expressions compared with untreated samples. C: untreated cells, TR: treatment by transfection reagent, S: treatment by scrambled control siRNA, siRNA: treatment by siRNA pool.

| Groups | P value | Significance |
| --- | --- | --- |
| GLA-C vs GLA-TR | 0.65 | Not statistically significant. |
| GLA-C vs GLA-S | 0.19 | Not statistically significant |
| GLA-C vs GLA-siRNA | 0.01 | Statistically significant |
| GLA-S vs GLA-siRNA | 0.003 | Statistically significant |
| RPL36A-C vs RPL36A-TR | 0.54 | Not statistically significant. |
| RPL36A-C vs RPL36A-S | 0.36 | Not statistically significant |
| RPL36A-C vs RPL36A-siRNA | 0.01 | Statistically significant |
| RPL36A-S vs RPL36A-siRNA | 0.002 | Statistically significant |
| HNRNPH2-C vs HNRNPH2-TR | 0.44 | Not statistically significant. |
| HNRNPH2-C vs HNRNPH2-S | 0.37 | Not statistically significant |
| HNRNPH2-C vs HNRNPH2-siRNA | 0.08 | Not statistically significant |
| HNRNPH2-S vs HNRNPH2-siRNA | 0.01 | Statistically significant |

1. **Supplementary Figure S1**

**Figure S1.** Knocking down the enhancer GH0XJ101390 by siRNA. (A) The enhancer GH0XJ101390 sequence mapped at genomic coordinates (GRCh38): chrX: 101,390,257-101,393,641 with size of 3.443 kb. The siRNA SR304146A sequence targeting GH0XJ101390 transcript is underlined and highlighted yellow (Fig. S1A); the siRNA (SR304146B) sequence targeting the middle part of *RPL36A* exon 2 sequence (blue) is underlined and highlighted yellow (Fig. S1A and B); the siRNA (SR304146C) sequence targeting a piece of *RPL36A* transcript composed of parts from untranslated exon-1 (91.7%) and exon-2 (Fig. S1A and B), the two segments are underlined and highlighted yellow. Human *RPL36A* NM_021029.6 mRNA transcript used in the analysis to identify the exon 1 and exon 2 in the genomic sequence of GH0XJ101390.

**(A)**

101390257 ……………………………………………………………………………………………………………………………………………………..CAGGTTTGCAAGGAAGG 101390273

101390274 GAAGAGCCGCTAGGTACTTTCTACTTCTAGGCTTGATGATTGCTATTACAGGAGCCAAACAATTTCATTGTCACT 101390348

101390349 TTTCAGAACCTAGAGCTGCTGACTTACTCCCTCAACAGAAGGGTGCTGCTTTAATGCCAATCATTGTTCCTTTTC 101390423

101390424 TTCAAAGTACTTAGTTCATAATTGCACATTTATAAGCCTATTAGATTACTGTACCTCTCCCCCGGAACAGTGCCT 101390498

101390499 GCCACATAATGGGCATCCAATAACCATTTGTTGAATGGACCAACTGGCCATGTGATAGATGAACTGGAACAATTG 101390573

101390574 GCTCCCTCCAGGTCTTCATTGAATTAGCAGTTTGCTGCGAAAGGTGTTCCCAGTGACCTTGAGGTGGAACAGGAA 101390648

101390649 TGGAACGTGGAGGTGGAGGACCCCTTCATTCGACCCTAGTCCTGGCTTCCCTCGGGGACGGGGAGGCCAGAGGAT 101390723

101390724 CTGGGAAAAACACTGCTCCCTTCACTGAACGCCACTACGTAGCAGAGTCCACCAAAAGAGATAAAGTGACGAAAC 101390798

101390799 GTTGAAAGCTGCGGGGCGGGAGGAAGTCGGGGTGAGTGGACCACCCTTTAGGCGGAGTCCTTCCTTAAGCTCCCG 101390873

101390874 CCCGCGGCTCCGAACTGAGTCCTCTCAGCCGCCCGAGGGCGCTGCGCTGAGCCTTACACTCTATGATTGCTCCTA 101390948

**SR304146A**A

101390949 CCGACTCCCATGAGGAAGTGCGATCG**GGAACCTCCTATATACTTCCGTTTG**CCTCGCGGTTT**CTTTCTTTCCGCG** 101391023

**SR304146C**A

101391024 **CCGATAGCGCTCACGCAAGCATG**GTAGGACTTGCTGGTGGGGGCCGAGTAACATCCAGCTTAATCTTTCCCCCCC 101391098

101391099 TTCGTCGCCCGTGCTATGCCGGGATGGGTCCAGGCTCCTGTGTGGACTCGATATATCAGAGCAACCCAATCTTGC 101391173

101391174 CGGGATCAACCCTAAAGGGACCGGGCTACGGGGCCAGGAATTGAAGTGATGGGTTCAAAGAGGTAGAGTTAGCCG 101391248

101391249 GGGGTGGAGTTAATGAGGTCTCTTCCCTCTTTGGGGCTCGACGGAGGGAGGAAGCTCTGCTTGAAGCACATGGGG 101391323

101391324 CTGGCCCACCCTGTAGTCAGAGGTAGCAAGTAATGAGGAGTCCTCCTGGAGTGCCTCAGCTTTAGCTGGGTAAGG 101391398

**SR304146C**A

101391399 TAGGGCGTTGTGCCATTGTGGTCAAATAACATTGCACGTTCTGAACTGTTTCTTTACTAG**GTTAACGTCCCTAAA** 101391473

**SR304146B**A

101391474 **ACCCGCCGGACTTTCTGTAAGAAGTGTGGCAAGCACCAACCCCATAAAGTGACACAGTACAAGAAGGGCAAGGAT** 101391548

101391549 **TCTCTGTACGCCCAGG**GTAAGATGGATTCCGCATAATTTGGTGTTAATGTGACATTTGTGTTGTAGAATAACATA 101391623

101391624 CGAGTCCGGGTCTCTCTCCCTCCACGCCTGGCTTGAGCGTTAATATTTCTGCTGCGAAATTAAGATAAAACCTGT 101391698

101391699 AAGACTTACTTGCTGATCTTCAGTATTTTATAACAAATACCAATTCGTTTTCCCAGGAAAGCGGCGTTATGACAG 101391773

101391774 GAAGCAGAGTGGCTATGGTGGGCAAACTAAGCCGATTTTCCGGAAAAAGGTGAGTGGTAGTTACTATTTGACGTT 101391848

101391849 TCCCAGTTAATTGCCGTAAGGATATGCACTTGTCTCTAGTCCACACACTTCATGATATAGGTATAGCGTTAGTTT 101391923

101391924 AGCGAAGTTTTCACTGCACTGATATATCTAGTAGGTGATGGAGCTGGGAATGCAACTCATGTCTGACTAGTCCAC 101391998

101391999 AATACTGCACTATTTCAGTGTTTACGATTTTTTATCCTTTCCCTTCTGAAGAGGCAAAAAATTGAGGAATGTGCC 101392073

101392074 CTGCTTTCCTAAGAACTGAAGTGTGAGTACACTGGTAAATCCTTTCATTTGCCTTGTTCCTTATCTGTCAATATG 101392148

101392149 TCTGAATCCTCGCTTGTTGGTTGCACTAAGAATTGTTCTGTTGTTTCTCATCACAGAAATCTGCAGTCAACTACC 101392223

101392224 TGTTCTCGTGAAGTCTTAAAACTCTTATAGAATAGCCATTTAGGCCTTTCTGCTAGCCTCCTGAATTCTGTATTC 101392298

101392299 TCAGGCTGAGCGAGTTTCTGTTTACTCTCAAACCTTAGGTGATTTGGCTAACTCTTAAAGTAATTAGCACGATGA 101392373

101392374 TTGGAACGGAGCATTCTCTCCAACACAGCATTTCTTTTGGCACTTTGCTTCTTGTGCAGTTTAGCTCCAGAAAGT 101392448

101392449 ATTAAGGAATGACTTTAGTGCTCATTTGGATGCAGTAAGTGGTTTGATCTCAGGGTGGCAAAAAGAATGCTTTTT 101392523

101392524 TTATACCTTTTCACATTCGGATAACTTGTTTAGAAGACAGAGGTTCTAACTAGGTTTTGGCCTATTAAGAACTGC 101392598

101392599 AAACTAGCAGCAGCAGAACTCTGGCTAAAGGGGCAAGCTTATTAGGAAATTGAGTATTTAAAAGTTGAGCTACCA 101392673

101392674 TATGATCCAACAATCCCACTGCTGGGTATATACCCAGAAGAAAATCGGTATATCAAAGAGATATCTGCACTCCTA 101392748

101392749 TGTTTGTTGTAGCACTGTTTATAATAGCTAAGATTTAGAAGCAACCTTAGTGTCCATCGGGATGAATGGATAAAG 101392823

101392824 AAAATGTACCTATACGCGGCCAGGCACGGTGGCTTGTGCCTAGCACTTTGGAAAGCCGAGGCGGGTGGATCACCT 101392898

101392899 GAGGTCAGGAGTTCGAGACCAGCCTGGCCAAGATAGTGAAACCCCGTCTCTAGTAAAAATACAAAAATTAGCCGG 101392973

101392974 GCTTGTGGTGTGGGCCTGTAATCTCAGCCACCCGGGAGGCTGAGGCAGGAGAATCGCTGGAACCTGGGAGGCAGA 101393048

101393049 GGCTGCAGTGAGCCGAGATCACGCCACTGTACTCCAGCCTGGGCGACAGAGCAAGACTCCATCTCAAAAAAAAAA 101393123

101393124 AAAAAAAAAAGGGAAAAAGAAAATGCACCTATACACAGTGGTACTATTCAGCCATAAAAAGAATGAGATCCAGTC 101393198

101393199 ATTTACAACAACATGGGTGGAACTGGAGATCGTTATGTTAAGTGAAATAGGCACACAAAGACAAGCATCACATGT 101393273

101393274 TCTTGTTTGTGGGATCTAAAAATCAAAACAAGTGGACTTGTCATATAGAGAGTAGAAGGATGGTTACCAGAAGCT 101393348

101393349 GAGAACTTCTGGTGGCGGGAGGTGGGGATGGTTAATGGGTACAAAAAGAAAAAAGAATGAATTAGACCAACTATT 101393423

101393424 TGATAGCACGACAGCGTGACTAAAGTCAATAACTTAGTTACATATTTTAAAATAACTTAGAGTGTAATTGGATTG 101393498

101393499 TTTGTACCTCAAAGAAAAAATGCAATAAAACTTTACAGTGGAGAAACCTAACAAGCACTACCTCAGCCAGGTAAT 101393573

101393574 CAAGGTTAACATCAACAGTCACGAGTCATGTTGATATATACCCTTGATAAGGTGTGATGAAAATGACA………………. 101393641

**(B)**


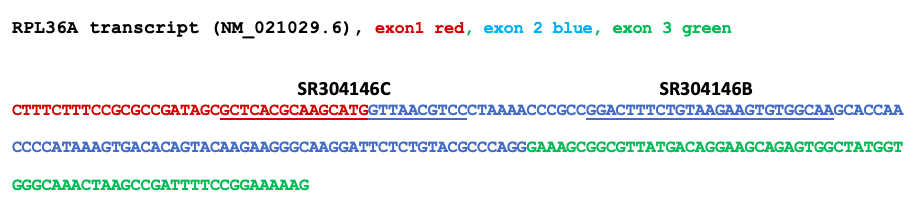

Supplement: Supplementary file 1 [file Table1.docx]
